# Minnesota State Dental Society

**Published:** 1884-11

**Authors:** 


### Minnesota State Dental Society.

The first annual meeting of the Minnesota State Dental Society was held in St. Paul, Wednesday, July 16th, 1884. The meeting was called to order at 10 o'clock a. m. by the President, Dr. H. M. Reid, of Minneapolis. The Recording Secretary, Dr. H. L. Crittenden, being absent, Dr. H. A. Knight, of Minneapolis, was appointed secretary protem. Reports of committees were called for, but none were yet ready to report. The roll being called, about twenty members responded and paid dues. On motion, Dr. D. R. Jennings, of Cleveland, Ohio, Dr. E. P. Call, of Peoria, Illinois, and Dr. H. Newington, of St. Louis, were elected honorary members. A cordial invitation was extended to all visiting dentists to take part in the discussion before the meeting.

Adjourned until 2 o'clock in the afternoon.

NOON SESSION.

The Association was called together at 2 o'clock by the President. Minutes of the morning session were read and approved. A letter was read from J. A. Chambers & Co., of St. Louis, Mo., asking that the Archives of Dentistry be made the official paper of their Association. The Association accepted and granted the request. Committee of Membership reported the names of Dr. Hale, of St. Paul; Dr. Whiting, of Northfield; Dr. Brown, of St. Paul, and Dr. Marden, of New Ulm, as candidates for membership. Upon a ballot being had, they were unanimously elected. Applications for membership were made by Dr. F. A. Williamson, of Red Wing, and Dr. Merrill, of Albert Lee. They were referred to the Committee on Membership. Dr. E. J. Miller, of St. Peter, then gave a very interesting paper on Regulating Teeth. He took for the basis of his discussion a very difficult case of irregularity, which he has successfully treated. After his paper a general discussion of the ideas which he had suggested ensued, in which quite a number took part. Dr. J. B. Lawrence, of New York, was elected an honorary member. Dr. H. L. Crittenden, of Northfield, read his paper on Articulation. The Doctor described the process of changing the articulation of teeth when they are misplaced in the mouth. He used models to illustrate the methods which he set forth, and the clearness of his demonstration was a subject of general comment. He closed with an appeal to the profession to go upward to new discoveries in dental science and reminded them that the nearer art approached Nature, the higher standard it assumed. Some time was occupied in asking and answering questions on the subject, which added much to a clearer understanding of points presented in the paper.

The following telegram was received from Milwaukee:

"Dr. H. M. Reid, President—The Wisconsin State Dental Association, in convention assembled, send greeting to our newly organized sister Society in Minnesota, with best wishes for its greatest possible usefulness.

W. F. Lewis,

Chas. C. Chittenden, } Committee."

The Secretary was instructed to acknowledge the receipt of the telegram, and return congratulations of the Association of Minnesota.

Adjourned until 9 o'clock to-morrow morning.

SECOND DAY—MORNING SESSION.

The Association was called to order at 10 o'clock by the President. The minutes of yesterday's meeting were read and approved. Dr. J. Taft, of Cincinnati, was elected an honorary member of the Association. Dr. J. L. Jacobs, of St. Paul, read a paper on Continuous Gum; giving his methods of working it. The discussion on this paper was deferred to another time. In the meantime the Doctor made preparation for practical demonstration of the process. Dr. W. N. Murray, of Minneapolis, now read a paper on Artificial Crowns, differing from the Richmond.

Discussion on this paper was also postponed, and the Association adjourned to L. W. Lyons' office in the Sherman Block, for clinical observations. Clinics were conducted by Dr. Lyons and Dr. Brown, of St. Paul: Dr. Jacobs also demonstrating the method of working continuous gum. Committee on Corporation reported that the constitution and by-laws of the Minnesota State Dental Association, had been filed in the office of the Secretary of State, and that the incorporation had been effected.

The Society consists of active and honorary members. Active members shall be 21 years of age, residents of the State, and of good moral character.

They shall have diplomas from reputable dental colleges, and shall have practiced five years. Active members are required to pay an entrance fee of $5.00, and annual dues, $3.00.

Adjourned.

The afternoon session was called to order at 3 o'clock. Some discussions were had on the subject of continuous gum; after which a discussion of artificial crowns followed, in which many members took part. Charges were preferred against Dr. Platon, of Fergus Falls, a member of the Association for advertising in an objectionable manner, which is especially prohibited by the Constitution. The charges were preferred by Drs. A. T. Smith, C. H. Goodrich, and J. B. Gillis. The Chair appointed a committee of investigation, consisting of three persons, who were to report at the next annual meeting. The Association now proceeded to the election of officers for the ensuing year, which being had, the following were found to be elected: President, Dr. L. W. Lyon, of St. Paul; Vice-President, Dr. F. A. Williams, of Red Wing; Recording Secretary, Dr. H. L. Crittenden, of Northfield; Corresponding Secretary, Dr. T. E. Martindale, of Minneapolis; Treasurer, Dr. A. T. Smith, of Minneapolis. The officers elect were now installed. Minneapolis was chosen as the place of the next annual meeting, the time being the third Wednesday in July, 1885.

On motion it was decided that the election and installation of officers be made the last order of business at each annual meeting, and that the President's address be the first order at the annual meeting succeeding the election. Dr. H. M. Reid then delivered his retiring address, the subject of which was "Dental Societies," in which he set forth very clearly many of the advantages and benefits which accrued to the dental profession, in association work. He regarded the question of a strong dental society of especial importance to the State of Minnesota. Laws have been passed in other States against quackery, and the result of this has been that Minnesota is overrun by poor dentists. He suggested the appointment of a committee of one from each congressional district to make effort for procuring the passage of the law regarding the practice of Dentistry in the State. The points presented were received with enthusiasm and were laid over for discussion.

Dr. Taft, of Cincinnati, read a report on diseased gums, and their treatment, upon which there was considerable discussion. The Society now adjourned till 7:30 p. m.

### EVENING SESSION.

The Association was called to order at eight by the President, Dr. Lyon. The subject of the retiring President's address was taken up for discussion. Quite an animated discussion ensued occupying about half an hour, when the following resolution was offered and adopted unanimously:

WHEREAS, At present there exist no laws in our State pertaining to the practice of Dentistry, and

WHEREAS, The Legislative bodies of this State will be this winter in sessi n.

THEREFORE, BE IT RESOLVED, That it is the sense of the Minnesota State Dental Society that such steps should be taken as shall be deemed best for securing laws regulating the practice of dentistry similar to those in operation in other States.

Dr. Taft gave a brief history of the operation of such legislation in Ohio, and other States, clearly showing the great good accruing, not only to the profession, but to the people at large, by such enactments. The remarks showed very clearly that such laws can be, and are executed wherever there is a will. On motion of Dr. G. W. Avery, of Minneapolis, a committee of five was appointed to report at the earliest time possible on some definite course of action. The Chairman appointed as this committee, Dr. G. W. Avery, Dr. A. T. Smith, Dr. H. M. Reid, of Minneapolis, and Dr. A. P. Welsh, of St Paul. Drs. Goodrich, Little, and Weeks were appointed Auditing Committee, with instructions to report as early as practicable. The Association now adjourned to meet to-morrow morning.

A session was not held in the evening, but upon invitation the members met at the Windsor Hotel for enjoyment of the banquet which had been prepared by the profession of St. Paul and Minneapolis for the Association. It was a delightful occasion. Dr. L. W. Lyon, of St. Paul, presided. Numerous toasts were given, and responses made in a quite happy manner. The evening was one of unusual pleasure and enjoyment.

### THIRD DAY—FRIDAY MORNING.

The Association was called to order at 10 o'clock, the President in the Chair. Minutes of the last session were read and approved. The Committee on Membership reported favorably on the names of Dr. Medd, of Owattona, Dr. Serle, of Owattona, Dr. Harris, of Lake City. A unanimous vote was taken, electing them to membership. Dr. J. H. Martindale, of Minneapolis, was instructed to respond immediately to the telegram of congratulation received from the Wisconsin State Dental Association.

Dr. J. B. Lawrence, of New York, then read a paper on the assimilation, which showed patient and careful study, and familiarity with the subject. A general discussion of the subject of the paper followed, which continued until 12 o'clock, when the society adjourned until evening.

AFTERNOON SESSION.

The Association met at 2 o'clock. The committee appointed to consider the matter of procuring legislation reported the following names as committee for this purpose: Dr. H. M. Reid, of Minneapolis, Chairman, Dr. L. M. Lyon, of St. Paul, Dr. A. M. Bailey, of Minneapolis, Dr. J. M. Welsh, of St. Paul, Dr. T. E. Weeks, of Minneapolis. It was on motion decided that a committee of two be appointed to assist the Secretary in obtaining a complete list of the dentists of the State, and their post office addresses. Dr. G. M. Dixon, of Winona, and Dr. H. A. Knight were appointed as this committee. The President announced the following committees for the ensuing year:

Executive Committee—H. M. Reid, Minneapolis; T. E. Weeks, Minneapolis; H. A. Knight, Minneapolis; C. E. Hale, St. Paul; J. L. Jacobs, St. Paul.

Committee on Membership—C. H. Goodrich, St. Paul; W. N. Crary, St. Paul; A. M. Dixon, Winona; J. E. Miller, St. Peter; D. C. St. John, Minneapolis.

Committee on Professional Conduct—J. H. Martindale, Minneapolis; J. M. Welsh, St. Paul; W. N. Murray, Minneapolis.

The following delegation was appointed to attend the annual convention of the American Dental Association, to be held in Saratoga the first of next month:

L. M. Lyon, St. Paul; H. M. Reid, Minneapolis; C. M. Bailey, Minneapolis; A. T. Smith, Minneapolis, J. E. Miller,

St. Peter; J. M. Welsh, St. Paul; J. B. Little, St. Paul; G. V. I. Brown, St. Paul.

The Auditing Committee reported that they had examined the Treasurer's accounts, and found them correct. Bills to the amount of $52.75 were presented and allowed. On motion of Dr. Reid, Drs. J. H. Martindale, H. A. Knight, and T. E. Weeks were appointed a Committee on Publication. The Association extended a request to the editor of the *Archives of Dentistry* to allow the paper of Dr. Crittenden, read at this meeting, to be published also in the *Dental Cosmos*, of Philadelphia.

The membership has increased about half, and now shows an enrollment of fifty.

The association then adjourned until the third Wednesday in July, 1885.

